# Sustainable Biopolymers for Environmental Applications: Advances and Future Perspectives Toward a Circular Economy

**DOI:** 10.3390/polym18050618

**Published:** 2026-02-28

**Authors:** Carlos A. Ligarda-Samanez, Mary L. Huamán-Carrión, Henry Palomino-Rincón, Fredy Taipe-Pardo, Elibet Moscoso-Moscoso, Domingo J. Cabel-Moscoso, Antonina J. Garcia-Espinoza, Dante Fermín Calderón Huamaní, Jackson M’coy Romero Plasencia, Jaime A. Martinez-Hernandez, Rober Luciano-Alipio, Jorge Apaza-Cruz

**Affiliations:** 1 Nutraceuticals and Biomaterials Research Group, Universidad Nacional José María Arguedas, Andahuaylas 03701, Peru; huamancarrionmary@gmail.com (M.L.H.-C.); hpalomino@unajma.edu.pe (H.P.-R.); ftaipe@unajma.edu.pe (F.T.-P.); emoscoso@unajma.edu.pe (E.M.-M.); 2 Environmental Engineering School, Universidad Nacional San Luis Gonzaga, Ica 11001, Peru; jesus.cabel@unica.edu.pe (D.J.C.-M.); antonina.garcia@unica.edu.pe (A.J.G.-E.); dante.calderon@unica.edu.pe (D.F.C.H.); jaime.martinez@unica.edu.pe (J.A.M.-H.); 3 Department of Mathematics and Physics, Universidad Nacional de San Cristóbal de Huamanga, Ayacucho 05000, Peru; jackson.romero@unsch.edu.pe; 4 Administrative Sciences Faculty, Universidad Nacional Autónoma Altoandina de Tarma, Junín 12731, Peru; rluciano@unaat.edu.pe; 5 Academic Department of Electronic Engineering, Universidad Nacional del Altiplano, Puno 21001, Peru; jlapaza@unap.edu.pe

**Keywords:** renewable biomaterials, natural polymer matrices, pollutant capture, green functionalization, environmental enhancement, applied sustainability

## Abstract

In recent years, sustainable biopolymers have attracted increasing attention in environmental engineering as alternatives to conventional synthetic materials due to their renewable origins, biodegradability, and functional versatility. However, their performance and technological viability are strongly influenced by structural design, modification strategies, and behavior under realistic environmental conditions. This review critically analyzes recent advances in biopolymers for environmental remediation, covering their main application formats such as hydrogels, membranes, beads, aerogels, and composites, their interaction mechanisms with contaminants, and their performance relative to conventional adsorbents. Particular emphasis is placed on emerging approaches, including advanced functionalization, integration with inorganic phases, and green synthesis technologies, which have significantly improved efficiency, selectivity, and operational stability. Despite these advances, key limitations persist, particularly regarding mechanical robustness, regenerability, reproducibility, and scalability, underscoring the need for standardized evaluation protocols in complex matrices. The role of biopolymers within circular economy frameworks is also examined, emphasizing their capacity to integrate material sustainability, resource recovery, and multifunctional environmental applications. Overall, sustainable biopolymers are positioned not only as substitutes for traditional materials but also as strategic platforms for the development of next-generation regenerative environmental technologies.

## 1. Introduction

Environmental pollution has intensified to the point that it has become a global problem affecting water, soil, and air. In aquatic systems, there are reports of the persistent presence of potentially toxic metals such as lead, cadmium, and arsenic, as well as industrial dyes and emerging pollutants, including antibiotics and perfluoroalkyl and polyfluoroalkyl substances (PFAS), which have high chemical stability, bioaccumulation potential, and long-term adverse environmental effects [1,2,3]. In soils, the accumulation of pesticides and heavy metals from agricultural and industrial activities compromises soil quality, reduces microbial biodiversity, and promotes the transfer of contaminants to crops and groundwater [4,5]. On the other hand, air pollution continues to worsen due to the sustained increase in carbon dioxide (CO_2_), the main greenhouse gas, as well as exposure to volatile organic compounds (VOCs), which pose a significant risk to human health, especially in indoor environments [6,7]. In addition, there is a growing accumulation of non-biodegradable plastics and microplastics, whose dispersion across aquatic, terrestrial, and atmospheric ecosystems highlights the limitations of linear production and consumption models and underscores the urgent need for materials and strategies that reduce their environmental persistence [8].

Faced with this scenario, conventional environmental treatment and remediation technologies have significant limitations, as they often depend on fossil-based materials, consume significant energy, or generate secondary waste during application. This situation has highlighted the need to develop sustainable solutions that reduce the environmental impact associated with pollution control processes. In this sense, an environmental technology can be considered truly sustainable when it has key characteristics such as low energy consumption, minimal generation of secondary waste, operational stability in real conditions, the possibility of regeneration and reuse, economic viability, and compatibility with the principles of the circular economy. In this context, renewable and biodegradable materials have emerged as promising alternatives due to their ability to remove or mitigate pollutants through physicochemical mechanisms, while also helping to reduce the environmental impacts associated with their production, use, and final disposal [9,10,11,12]. In particular, bio-based materials, derived from renewable resources and proposed as substitutes for conventional petrochemical polymers, contribute to more sustainable environmental management by reducing dependence on fossil-based raw materials and decreasing the environmental persistence of conventional plastic waste [12,13].

In this context, sustainable biopolymers have gained increasing interest as functional materials for environmental applications due to their renewable origin, biodegradability, and structural versatility. In general, these materials can be classified into two main groups: (i) natural biopolymers, extracted directly from biological sources, and (ii) synthetic biopolymers of renewable origin, obtained through polymerization processes from monomers derived from biomass. Among natural biopolymers, polysaccharides such as starch and cellulose stand out, as well as proteins of plant or animal origin, which have a high density of functional groups, such as hydroxyls, carboxyls, and amino groups, favoring interactions with contaminants through physical, physicochemical, and, in some cases, chemical adsorption mechanisms [10,14,15,16,17,18,19]. On the other hand, renewable synthetic biopolymers, such as polylactic acid (PLA) and polyhydroxyalkanoates (PHA), are characterized by greater controllability of their mechanical properties, good processability, and structural stability, which broadens their applicability in environmental remediation systems, functional supports, and composite materials with a sustainable approach [11,12,20,21]. This differentiation is key to understanding the potential of sustainable biopolymers as alternatives to conventional polymers for pollution control and mitigation strategies, due to their renewable origin, reduced environmental persistence, inherent biodegradability, and the abundance of functional groups that enhance interactions with contaminants. Moreover, their capacity for chemical modification and the formation of hybrid materials enables improved selectivity, regeneration, and operational performance in environmental remediation processes.

From a circular economy perspective, sustainable biopolymers represent a strategic alternative for closing material cycles by integrating the use of renewable sources, their application in environmental solutions, and responsible management at the end of their useful life. Unlike conventional polymers, these materials can be obtained from biomass and agro-industrial by-products, used in environmental remediation processes, and finally reincorporated into natural or productive cycles through biodegradation, composting, or recycling, thus reducing the accumulation of persistent waste and dependence on fossil resources [11,22,23]. This comprehensive approach reinforces the role of biopolymers as functional platforms aligned with the principles of sustainability and the circular economy [12,13].

Unlike previous reviews, this study adopts an integrated approach that simultaneously integrates the types of biopolymers, their structural configurations, forms of application, and functional performance across different environmental compartments (water, soil, air, and waste management). It also incorporates analysis from a circular-economy perspective, linking material origins, their use in remediation systems, and their regeneration or end-of-life pathways. The overall objective of this review article is to critically analyze recent advances in the development and application of sustainable biopolymers. In particular, the following specific objectives are proposed:

1. Identify the main sustainable biopolymers used in environmental applications.
2. Analyze the mechanisms of interaction between biopolymers and different environmental pollutants.
3. Evaluate the primary forms of application and their performance in environmental systems.
4. Review the applications of biopolymers in water treatment, soil remediation, atmospheric pollutant capture, and waste management.
5. Discuss recent advances, implications from the circular economy, and prospects for these materials.

**Figure 1:**
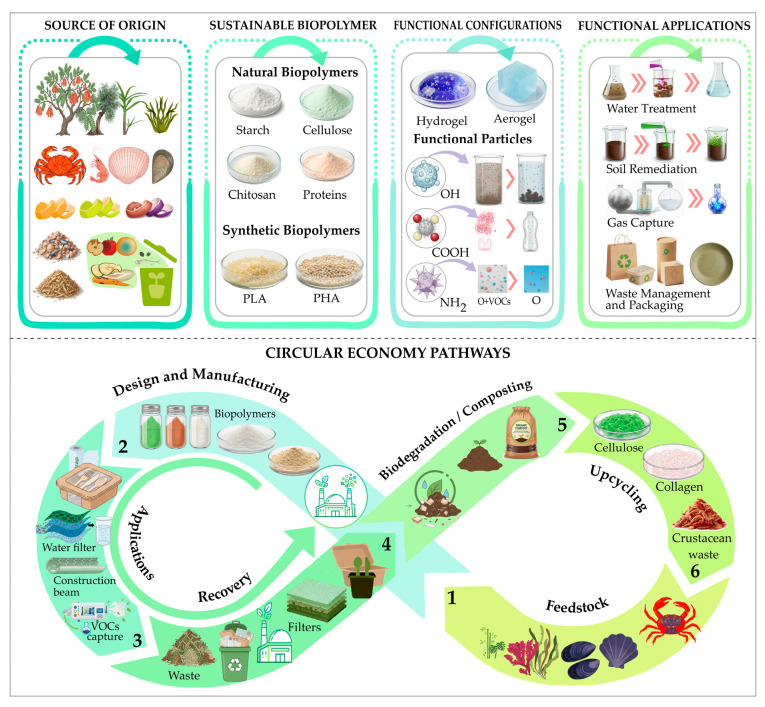
Conceptual framework of sustainable biopolymers for environmental applications in the context of the circular economy.

Figure 1 presents a conceptual map that integrates the relationships between the source of origin, the type of biopolymer, its form of application, environmental applications, and associated circularity routes.

## 2. Narrative Review Methodology

This review was conducted as a critical and integrative narrative review, following a structured and sequential methodological approach involving literature search, data refinement, study screening, and critical analysis [24,25,26], to provide an updated, comparative, and analytical overview of recent advances in the development and application of sustainable biopolymers for environmental applications within the framework of the circular economy. The objective of this methodological approach was not exhaustiveness, but rather the identification and critical analysis of representative studies with demonstrated technological and environmental relevance, integrating knowledge from different disciplines, including environmental engineering, materials science, and sustainability. The study was not conceived as a systematic review or a scoping review, but as a critical narrative synthesis based on a reasoned and intentional selection of the state of the art.

The literature search was carried out in a first stage using Scopus, Web of Science, PubMed, and Google Scholar databases. It was subsequently complemented by direct searches in recognized scientific publishers, including MDPI, Elsevier, SpringerLink, Taylor & Francis, and Wiley. The analysis period was explicitly delimited to publications between 2018 and 2026, prioritizing recent studies that reflect technological advances, emerging approaches, and current trends in the use of sustainable biopolymers, with particular emphasis on works addressing environmental applications under operationally relevant conditions.

The search strategy included combinations of keywords such as sustainable biopolymers, biopolymers, environmental applications, water treatment, soil remediation, adsorption, membranes, hydrogels, circular economy, biodegradable polymers, PLA, PHA, chitosan, cellulose, nanocellulose, and renewable polymers. The PRISMA or PRISMA–ScR guidelines were not formally applied due to the narrative nature of the review; however, a transparent narrative screening approach was adopted, respecting principles of methodological coherence, traceability of the selection process, and critical evaluation of the literature, as commonly recommended for integrative narrative academic reviews.

After removing duplicate records and conducting an initial screening based on titles and abstracts, inclusion criteria were applied, considering thematic relevance, methodological rigor, clarity in material characterization, and, most importantly, the relevance of the reported results to real environmental applications, including discussion of operational parameters, material stability, regeneration behavior, or scalability potential. Studies with insufficient methodological information, those not directly related to environmental applications, works focused exclusively on mechanical properties without a functional environmental link, and purely theoretical studies without experimental validation or without a critical discussion of practical applicability were excluded.

The finally selected articles were analyzed comparatively and organized according to biopolymer type, application form (hydrogels, particles, membranes, films, among others), target environmental compartment (water, soil, air, or waste), and their potential contribution to circular economy strategies. This organizational scheme is part of the review’s analytical framework, as it enabled the identification of technological advances, current limitations, technology readiness levels, and knowledge gaps, facilitating a critical discussion of future research directions and the scalability and feasibility of these materials in real environmental contexts.

## 3. Sources, Types, and Functional Characteristics of Sustainable Biopolymers

Sustainable biopolymers are materials derived from renewable resources that can degrade under environmental conditions, offering potential alternatives to conventional hydrocarbon-based polymers. These macromolecules are typically classified according to their origin into natural polysaccharides, proteins, and gums, as well as synthetic biopolymers obtained from biological resources, each with particular structural and functional characteristics that determine their performance in environmental and sustainable engineering applications. For example, polysaccharides have a high abundance of hydroxyl and carboxyl groups that facilitate electrostatic and hydrophobic interactions with organic and inorganic contaminants. At the same time, protein-based biopolymers (such as gelatin, casein, and plant proteins) and natural polysaccharide gums (such as gum arabic, xanthan gum, and alginates) can form three-dimensional matrices with a high density of active sites that enable adsorption, chelation, or encapsulation of target molecules, including heavy metals, dyes, and other emerging contaminants [27,28].

On the other hand, biodegradable synthetic biopolymers such as PLA and PHA combine the sustainability of their origin with mechanical and thermal properties that favor their processing in membranes, films, and composite structures. However, their additional functionalization remains an active area of research to improve their performance in contaminant remediation and separation [29,30,31,32]. Thus, the selection of a family of biopolymers for environmental applications depends on a balance between their functional characteristics and physical properties. While the former determines parameters such as affinity, selectivity, and interaction capacity with contaminants, the latter govern their structural stability, processability, mechanical resistance, and performance under real operating conditions. Simultaneously optimizing both sets of properties is essential to ensure their effectiveness and operational feasibility, as discussed in the following subsections.

### 3.1. Polysaccharides

Polysaccharides constitute the most widely investigated family of biopolymers for environmental applications due to their high natural availability, low cost, biodegradability, and high density of functional groups, including hydroxyl, carboxyl, and amino groups, which facilitate electrostatic interactions, hydrogen bonding, and complexation processes with organic and inorganic contaminants in water and soil. These structural characteristics, combined with their capacity for chemical and physical modification through methods compatible with green chemistry principles, have enabled the design of polysaccharide-based materials, such as hydrogels, beads, membranes, and composite microstructures, aimed at the adsorption, coagulation–flocculation, and separation of contaminants in wastewater treatment and environmental remediation processes. Recent reviews highlight advances in hydrogels and adsorbents derived from polysaccharides such as starch, cellulose, and chitosan, emphasizing their efficiency in capturing heavy metals, dyes, and antibiotics, as well as modification strategies to address limitations in solubility, mechanical stability, and regenerability for real-world applications [33,34,35].

Starch is one of the most abundant, economical, and renewable polysaccharides in nature, composed mainly of amylose and amylopectin, which gives it multiple hydroxyl groups available for interactions with contaminating species in aqueous solutions. This functional accessibility, together with the ease of chemical modification (e.g., introduction of carboxyl or amino groups through cross-linking or copolymerization), has enabled the development of starch-based adsorbents and composite materials with high removal efficiency for heavy metals and other emerging contaminants. Recent reviews report that starch-derived adsorbents, both native and modified, exhibit competitive adsorption capacities and improved performance against organic and inorganic contaminants when their structure and porosity are optimized, positioning them as promising materials for sustainable environmental remediation strategies [36].

Cellulose and its nanometric derivatives (nanocellulose) stand out for their large specific surface area, high porosity, and abundance of hydroxyl groups that facilitate interactions with metal ions, dyes, and other contaminants in aquatic media. These physicochemical characteristics, together with the possibility of surface modification (e.g., oxidation, functionalization, or assembly into hydrogels and membranes), have driven their application in adsorption and separation systems, including aerogel-type materials, sponges, and high-performance filtration membranes. The most recent studies demonstrate that nanocellulose-based materials can be engineered with highly accessible, functionalized structures that significantly enhance contaminant capture capacity compared to conventional microcrystalline cellulose, thereby consolidating their role in emerging wastewater treatment technologies [37,38].

Chitosan, derived from the deacetylation of chitin, is characterized by a high availability of protonatable amino groups in its molecular backbone, which confers a strong affinity for negatively charged metal ions and anionic dyes in aqueous solutions. This functional richness translates into a remarkable adsorption capacity, particularly when employed in cross-linked forms, such as hydrogels and nanofibers, or in combination with other polysaccharides, which enhance the accessibility of active sites. Specific reviews on chitin/chitosan systems show that these materials are not only biodegradable and biocompatible, but that their electrostatic interactions and surface chemistry make them particularly effective for the removal of heavy metals and dyes. Furthermore, they are amenable to additional modifications to optimize their selectivity and regenerability in environmental applications [39,40].

### 3.2. Proteins and Natural Gums

Proteins and natural gums have acquired a central role in the development of sustainable biopolymers for environmental applications due to their biodegradability, biocompatibility, renewable availability, and functional versatility. These biopolymers exhibit chemical structures rich in functional groups (–NH_2_, –COOH, –OH), which confer a high capacity for interaction with contaminants, encapsulation of active compounds, and the formation of three-dimensional networks suitable for adsorption, coagulation, and flocculation processes in aqueous systems [41,42,43,44].

Gelatin, obtained by partial hydrolysis of collagen, stands out for its ability to form thermoreversible gels, its high water solubility, and its aptitude to form electrostatic and hydrogen-bonding interactions with bioactive compounds and contaminants [45,46,47].

Several studies have demonstrated that gelatin-based matrices can be used effectively to microencapsulate phenolic compounds, antioxidants, and antimicrobial agents, thereby improving their stability under adverse environmental conditions, such as pH variations, temperature, and ultraviolet (UV) radiation [48,49]. In environmental applications, gelatin has also been evaluated as an adsorbent for heavy metals and dyes, either in the form of hydrogels, microspheres, or composite materials, showing removal capacities comparable to those of synthetic adsorbents [46].

Collagen, in turn, exhibits a highly organized fibrillar structure that allows chemical and physical modification to improve its mechanical resistance and stability in aqueous environments. Modified and cross-linked collagen materials have been employed as biosorbents for metal ions and organic contaminants, demonstrating the potential of these proteins as functional materials in water treatment strategies under a circular economy framework [50,51].

Natural gums and polysaccharides, such as tara gum (*Caesalpinia spinosa*), nopal mucilage (*Opuntia* spp.), and marine-derived alginates, constitute a sustainable alternative to synthetic coagulants and flocculants conventionally used in water treatment processes [52,53,54]. Tara gum is a non-ionic galactomannan characterized by high viscosity and the ability to form stable solutions at low concentrations. Recent research has evidenced its potential as a natural flocculant, mainly when used alone or in combination with other biopolymers, promoting the aggregation of colloidal particles and turbidity removal in wastewater [54,55]. Nopal mucilage, rich in complex polysaccharides and charged functional groups, has been extensively studied as a natural biocoagulant, demonstrating remarkable efficiency in removing suspended solids, dyes, and heavy metals. Its use is particularly attractive in rural communities and regions with limited access to conventional chemical products, due to its low cost, local availability, and minimal generation of toxic byproducts [52,56]. Alginates, linear copolymers of β–D–mannuronic acid and α–L–guluronic acid, possess a high gelation capacity in the presence of divalent cations, making them ideal materials for simultaneous flocculation and encapsulation processes. Modified alginates have been successfully employed for immobilizing adsorbents, microorganisms, and active compounds, thereby enhancing contaminant removal efficiency and facilitating material recovery after treatment [57,58]. Taken together, the use of natural proteins and gums such as gelatin, collagen, tara gum, nopal mucilage, and alginates reinforces the development of sustainable environmental solutions. Their relevance can be assessed within a framework based on key circular economy principles, including renewable resource utilization, the potential for biological byproduct valorization, material recirculation or regeneration capacity, and environmentally compatible end-of-life behavior. Under these criteria, these biopolymers emerge as promising functional platforms for environmental applications, combining natural availability, structural versatility, and tunable properties that enhance their performance in contaminant remediation and pollution control processes.

### 3.3. Synthetic Renewable Biopolymers

Polylactic acid (PLA) and polyhydroxyalkanoates (PHA) are biodegradable synthetic biopolymers obtained from renewable resources such as fermentable sugars and vegetable oils, which have gained relevance in environmental and agro-industrial applications. PLA, mainly produced by the polymerization of lactic acid, is characterized by good processability, transparency, and mechanical strength, which has favored its use in biodegradable packaging, films, and membranes for separation and encapsulation processes [59,60,61,62]. In turn, PHAs constitute a family of microbial polyesters synthesized intracellularly by various bacteria as carbon and energy storage materials. These polymers exhibit high biodegradability in natural environments and tunable properties that depend on their monomer composition, making them attractive materials for the development of membranes, microbial immobilization supports, and controlled-release systems for environmental applications [63,64]. Both PLA and PHA represent sustainable alternatives to conventional petroleum-derived polymers, contributing to reducing environmental impact and advancing strategies aligned with the principles of the circular economy.

## 4. Forms of Application and Functional Configurations in Environmental Engineering

In recent decades, research on sustainable polymeric materials has experienced significant growth, focusing not only on the development of new biopolymers but also on optimizing their functional configurations for environmental engineering applications with reduced environmental footprint and high operational efficiency. Materials such as hydrogels, aerogels, and porous polymeric structures have demonstrated unique properties in water retention, high specific surface area, and contaminant adsorption capacity, making them ideal candidates for the remediation of contaminated water and soils. Likewise, sustainable biopolymer-based membranes enable ultrafiltration (UF) and nanofiltration (NF) processes that enhance the separation of organic and inorganic species, with lower waste generation and greater biocompatibility. On the other hand, biodegradable films and coatings extend the use of biopolymers to environmental barrier applications and plastic waste mitigation, contributing to an integrated circular economy. These advances reflect the interconnection between material design and environmental functionality, promoting clean and efficient technologies for contaminant management [13,65,66].

### 4.1. Hydrogels, Cryogels, and Aerogels

Hydrogels, cryogels, and aerogels are versatile classes of polymeric materials with highly porous three-dimensional structures that confer a unique combination of high water retention, large specific surface area, and strong affinity for diverse contaminant species. This porosity facilitates the interaction and capture of metal ions and dyes present in aqueous effluents. At the same time, the macroporous matrices of cryogels enhance mass transport and reduce resistance to water flow, which is beneficial for continuous adsorption applications. Aerogels, in turn, stand out for their extremely high surface area and low density. These characteristics promote the physical and chemical adsorption of organic and inorganic contaminants with greater efficiency. Collectively, these materials have been the subject of increasing research within environmental engineering, where efforts are focused on optimizing their structural and functional properties for applications in wastewater treatment, heavy metal remediation, and the removal of organic contaminants, prioritizing approaches that integrate sustainability and adsorption efficiency in advanced purification processes [67,68,69].

### 4.2. Beads and Functionalized Particles

Functionalized beads and particles have become established as a highly effective configuration for water treatment, enabling integration of chemical design (functionalization) with operational performance (batch or flow handling). In particular, the use of biopolymeric matrices (e.g., alginate/chitosan and their hybrids) facilitates the immobilization of active phases (e.g., chelating agents, nanostructures, magnetic components, etc.) within a macroscopic support, thereby enhancing selectivity toward contaminants while avoiding the practical problem of recovering fine powders after treatment. Moreover, targeted functionalization increases the density of active sites and can promote combined mechanisms (complexation, ion exchange, electrostatic interactions). At the same time, material reusability (through adsorption–desorption cycles) becomes a key criterion for sustainability and scalability. These trends are widely discussed in recent reviews on polysaccharide-based beads for water purification and, at the applied level, are evidenced in the design of composite beads capable of simultaneously removing metals and dyes with stability and performance under complex matrix conditions [70,71,72].

### 4.3. Sustainable Membranes

Sustainable membranes have gained prominence as separation platforms for ultrafiltration and nanofiltration by enabling integration of process performance with strategies to reduce environmental impact. In this context, techniques such as electrospinning facilitate the production of nanofibrous layers with tunable architectures (porosity, interconnectivity, and thickness), while casting/phase inversion and hybrid approaches enable the scalable fabrication of membranes with controlled microstructure and specific surface functionality. In addition, the incorporation of bio-based nanocomposites (e.g., reinforcements derived from cellulose/chitosan or polymeric networks of natural origin) is used to enhance stability, fouling resistance, and contaminant affinity, guiding membrane design toward more durable systems compatible with circularity-oriented approaches (renewable materials, reduced toxicity, and improved end-of-life management) [73,74].

### 4.4. Biodegradable Films and Coatings

Biodegradable films and coatings constitute a key strategy for reducing dependence on conventional plastics in packaging and environmental barrier applications, while simultaneously providing functional properties such as protection against oxygen, moisture, and microbial agents. Recent advances show that these materials, derived from natural biopolymers (polysaccharides, proteins) and biocomposites reinforced with nanometric fillers, are not only biodegradable and renewable but can also be engineered to extend the shelf life of sensitive products (such as fresh foods) and reduce post-consumer waste, thereby aligning with circular economy objectives. Furthermore, current research highlights the incorporation of bioactive components, multilayer structures, and smart systems that expand their functions beyond simple packaging, offering enhanced barrier properties, antimicrobial activity, or responsiveness to environmental stimuli. These developments open the door to integrated environmental applications ranging from sustainable packaging systems to functional protective coatings [75,76,77].

Despite the considerable progress reported in recent studies, the performance of biopolymer-based systems remains highly context-dependent, as adsorption capacity, selectivity, and stability are strongly influenced by experimental conditions, matrix composition, and operational parameters. Consequently, direct comparisons among studies should be interpreted cautiously, since variations in methodology, testing environments, and regeneration protocols often lead to inconsistencies in reported efficiencies. These factors highlight the importance of comparative analyses that consider not only peak performance values but also robustness, reproducibility, and applicability under realistic environmental conditions.

To systematize and compare the main functional configurations based on biopolymers used in environmental engineering, Table 1 summarizes the most representative application forms described in this section, including hydrogels/cryogels/aerogels, beads and functionalized particles, sustainable membranes, and biodegradable films or coatings. In particular, their main operational advantages, technical limitations, and representative performance examples are contrasted, providing an integrated overview that facilitates evaluation of their suitability for specific environmental applications, operating conditions, and sustainability criteria. This comparison enables identification of trends, strengths, and challenges associated with each configuration. It serves as a basis for discussing their scalability and potential for implementation in real environmental treatment and remediation systems. It should be noted that the performance values compiled from different studies are not strictly comparable, as variations in experimental conditions, including pH, ionic strength, competing ions, contact time, and regeneration protocols, may significantly influence reported adsorption capacities and removal efficiencies.

The functional configurations based on biopolymers analyzed in this section demonstrate that the environmental performance of these materials does not depend solely on their chemical composition, but rather on the interaction among their structural architecture, the capture or separation mechanisms involved, and the actual operating conditions. Highly porous structures, such as hydrogels, cryogels, and aerogels, promote adsorption and contaminant retention. In contrast, configurations such as beads, membranes, and films offer additional advantages in process control, material recovery, and potential integration into continuous systems, because their defined geometry, mechanical stability, and structural integrity facilitate controlled flow regimes, allow easier separation of the material from treated media, and enable implementation in fixed-bed, filtration, or modular treatment units commonly used in industrial-scale operations. These functional differences determine their suitability for specific applications and highlight the need to select the most appropriate configuration according to the type of contaminant, the environmental compartment, and sustainability and scalability criteria. On this basis, the following section explicitly addresses the environmental applications of biopolymers, examining in depth their performance in water treatment, soil remediation, the capture of atmospheric pollutants, and waste reduction.

## 5. Environmental Applications of Biopolymers

Biopolymers have emerged as functional materials of growing interest in a wide range of environmental applications due to their renewable origin, biodegradability, structural versatility, and ability to interact with contaminants across different environmental compartments. Their potential lies not only in replacing conventional fossil-fuel–based materials but also in providing solutions to pollution problems in water, soil, and air, as well as in waste reduction, owing to their physicochemical properties, eco-compatibility, and more sustainable manufacturing processes. Given the applied nature of biopolymers, the evaluation of their environmental applications must consider not only their efficiency under controlled conditions, but also their performance in real matrices, long-term stability, regeneration potential, and integration within sustainability and circular economy strategies—issues that have received increasing attention in recent literature [12,107,108,109].

### 5.1. Water Treatment

Water treatment using biopolymers encompasses a wide range of contaminants, including heavy metals, industrial dyes, and persistent organic compounds, whose interaction mechanisms depend on both the nature of the contaminant and the complexity of the aqueous matrix. Recent literature consistently indicates that the performance of these materials is strongly conditioned by factors such as pH, ionic strength, the presence of natural organic matter, and the coexistence of competing ions, which generate significant differences between results obtained in synthetic solutions and those observed in real effluents. For this reason, more robust evaluations tend to report ranges of removal efficiencies and adsorption capacities rather than isolated values, better reflecting representative operating conditions. Likewise, critical challenges for large-scale implementation include material regeneration and reuse, efficiency losses due to ionic competition, and limitations in process scalability, particularly in continuous systems and industrial applications [44,65].

### 5.2. Soil Remediation

In the remediation of contaminated soils, the use of biopolymers has primarily focused on contaminant immobilization rather than complete removal, aiming to reduce bioavailability and associated environmental risks from heavy metals and persistent organic compounds. This approach is particularly relevant in agricultural soils and sensitive ecosystems, where total contaminant extraction is not always feasible or desirable. Recent studies highlight that the long-term stability of biopolymeric systems is a critical factor, as processes such as pH fluctuations, wet–dry cycles, and material aging can affect contaminant retention and generate risks of secondary mobilization. Likewise, interactions with soil microbiota and the fundamental physicochemical properties of soils (texture, organic matter content, biological activity) play a determining role in the performance of these materials, influencing both their effectiveness and their collateral effects on soil quality. Therefore, recent literature emphasizes the need to evaluate biopolymers under representative field conditions and over extended temporal scales, prioritizing indicators of stability, environmental safety, and ecological compatibility [110,111,112,113].

### 5.3. Gas Capture

Applications of biopolymers for the capture of gaseous contaminants have focused primarily on selectivity for CO_2_ and volatile organic compounds (VOCs), rather than on absolute adsorption capacity, as this criterion is decisive for their viability in real-world applications. In the case of CO_2_, recent studies highlight the use of functionalized biopolymeric matrices, particularly those based on cellulose, chitosan, and hybrid systems containing amine groups, where selectivity and stability over successive adsorption–desorption cycles constitute critical evaluation parameters. Similarly, for VOC mitigation, the performance of biopolymeric materials is analyzed under dynamic flow conditions that simulate industrial or indoor air environments, since static assays tend to overestimate real efficiency. Nevertheless, despite the reported advances, the literature agrees that biopolymers still present limitations compared to conventional inorganic materials, particularly in terms of thermal stability, mechanical resistance, and operational durability, which restrict their application to specific niches or hybrid systems rather than direct replacement of established technologies [114,115].

### 5.4. Waste Management and Packaging

Waste management and the design of sustainable packaging represent one of the most critical domains in which biopolymers offer a potentially significant contribution compared to conventional fossil fuel–derived polymers, by reducing the accumulation of persistent plastic waste and the overall environmental footprint of product life cycles. The assessment of the net environmental impact of bioplastics, particularly materials such as polylactic acid (PLA) and polyhydroxyalkanoates (PHA), typically considers not only greenhouse gas emissions and primary energy use, but also human toxicity and ecotoxicity associated with each stage of their life cycle, from production to end-of-life, through methodologies such as Life Cycle Assessment (LCA), which enables systematic comparison with traditional fossil-based plastics. Beyond potential advantages in terms of carbon footprint, the end-of-life of biopolymers, including routes such as industrial composting, controlled biodegradation, or recycling, introduces operational complexities that depend on available infrastructure and specific environmental conditions, thereby conditioning the actual substitution of fossil plastics and their integration into circular economy strategies. In this context, recent studies indicate that while biopolymers can reduce dependence on fossil-based materials and mitigate specific environmental impacts, their effectiveness as true substitutes is strongly influenced by factors such as the efficiency of waste collection and treatment systems, biodegradation rates under uncontrolled conditions, and emissions associated with production, which demands a critical and contextualized evaluation [12,116,117,118,119].

**Figure 2:**
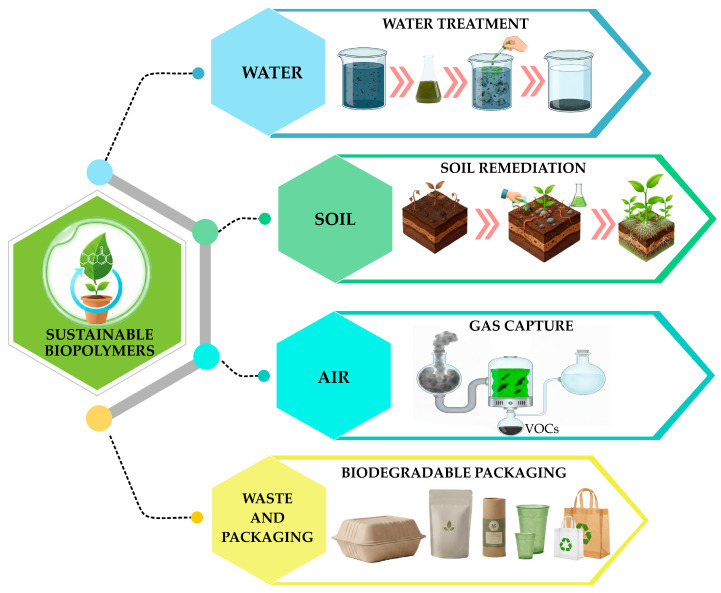
Application pathways of biopolymers in environmental compartments: water, soil, air, and waste management systems.

Overall, these findings indicate that although biopolymers demonstrate broad functional potential across environmental compartments, their practical implementation depends on context-specific optimization, technological readiness, and integration with existing treatment infrastructures. To schematically synthesize the main application pathways of biopolymers in environmental engineering, Figure 2 illustrates the domains in which these materials have demonstrated the most significant functional relevance, including water treatment, soil remediation, gaseous pollutant capture, and waste management through sustainable packaging. This schematic enables visualization of the relationships among different environmental compartments and the most representative uses of biopolymers, highlighting their versatility as material platforms for addressing diverse environmental challenges through an integrated approach aligned with sustainability and circular-economy principles.

## 6. Recent Advances and Emerging Technologies in Sustainable Biopolymers

Research on sustainable biopolymers has evolved toward the development of emerging technologies to improve functional performance, process sustainability, and applicability under real environmental conditions. This evolution is reflected in the increasing emphasis on advanced biopolymer functionalization, the integration of green processes, and the design of materials with more complex architectures and responsive behaviors, capable of overcoming previously identified limitations in applications such as water treatment, soil remediation, atmospheric pollutant capture, and waste management. In this context, biopolymers are no longer regarded as passive materials. However, they are instead conceived as advanced functional platforms, whose development directly addresses the challenges of efficiency, regenerability, and scalability discussed previously, in alignment with the principles of sustainability and circular economy that underpin current environmental strategies [11,12,65,72].

After establishing the conceptual framework for emerging technologies in sustainable biopolymers, one of the most prominent research directions is the functionalization of nanocellulose by incorporating inorganic and structural agents that enhance its physical, chemical, and functional properties. Nanocellulose, due to its high aspect ratio, large specific surface area, and abundant surface hydroxyl groups, constitutes an ideal matrix for the formation of hybrid materials with clays, metallic nanoparticles, and metal–organic frameworks (MOFs), enabling improvements in mechanical strength, adsorption capacity, contaminant selectivity, and structural stability of biopolymers in environmental and functional applications. In particular, the combination of nanocellulose with MOFs has shown great potential for developing adsorbents and multifunctional materials with tunable porosity and a wide range of applications, from CO_2_ capture to gas separation and water treatment, due to the structural and chemical synergy between the two components [120,121]. Similarly, integrating nanocellulose with metallic or oxide nanoparticles yields hybrid systems with enhanced properties, such as catalytic activity and antimicrobial activity, expanding their application potential beyond that of conventional biopolymers [122]. Finally, the incorporation of clays and other nanoreinforcements into nanocellulose matrices has been shown to significantly improve the mechanical and thermal properties of composites, contributing to the development of more robust and versatile bio-based materials capable of addressing applied challenges in environmental engineering and sustainable processes [123].

In line with advances in hybrid materials, another central axis of emerging technologies in sustainable biopolymers is the adoption of green synthesis and modification processes to reduce environmental impact and improve overall process efficiency. Among the most relevant strategies are ultrasound- and microwave-assisted techniques, which enable intensified mass transfer, reduced processing times, and lower energy consumption compared to conventional methods, while also promoting improved functionalization and structural control of biopolymeric matrices [124,125]. Complementarily, ionic liquids (ILs) have been extensively investigated as alternatives to traditional organic solvents, particularly for the processing and dissolution of biopolymers such as cellulose, owing to their low volatility, high solvating capacity, and recyclability [126]. More recently, natural deep eutectic solvents (NADES) have emerged as an even more sustainability-aligned option, as they are formulated from naturally derived compounds, exhibit low toxicity, and enable their integration in both extraction processes and the functional modification of biopolymers [127,128]. Taken together, these green technologies not only reduce the environmental footprint associated with material production but also enhance the performance and viability of biopolymers in real-world applications.

In addition to material functionalization and the incorporation of green processes, innovative biopolymer-based materials constitute one of the most relevant advances in the new generation of emerging technologies, due to their ability to respond in a controlled manner to external stimuli and to operate effectively in complex environments. In particular, responsive hydrogels, defined as polymeric networks capable of reversibly modifying their structure and properties in response to variations in pH, temperature, electric fields, or the presence of contaminants, have attracted growing scientific interest. These characteristics enable adaptive responses applicable to controlled release of active agents, selective contaminant capture, and functional signaling in environmental processes. Recent reviews have systematized these developments, highlighting the diversity of response mechanisms and their implications for environmental and biomedical applications, including the removal of heavy metals and phosphates from contaminated waters and the design of intelligent actuation systems [129]. Complementarily, biopolymer-based environmental biosensors and their composites have gained relevance as promising tools for real-time contaminant monitoring, integrating renewable biopolymeric materials with electrochemical platforms that enhance device sensitivity, selectivity, and sustainability compared to conventional technologies [130]. These approaches reflect a transition from passive materials to intelligent, multifunctional systems capable of adapting and responding in situ to environmental changes and providing innovative solutions to monitoring and remediation challenges.

From an applied perspective, recent advances in sustainable biopolymers necessitate situating their performance relative to conventional materials widely used in environmental engineering, such as activated carbon, zeolites, and synthetic membranes. These inorganic materials have historically demonstrated high removal efficiency, structural stability, and operational robustness, leading to their adoption at the industrial scale for adsorption, separation, and water treatment. Nevertheless, the growing interest in functionalized biopolymers is not limited to point efficiency but also incorporates additional criteria, such as biodegradability, a lower environmental footprint, functional versatility, and regeneration potential, which are particularly relevant in complex environmental systems. In this context, the performance of biopolymers critically depends on the functionalization strategy, operating conditions, and the nature of the treated matrix, reinforcing the need for comparative evaluations based on ranges of efficiency, selectivity, regenerability, and scalability, rather than isolated direct comparisons, and orienting the analysis toward complementarity approaches and the design of hybrid solutions [65,66,72,130,131].

To synthesize the main emerging technological approaches described in this section, Table 2 summarizes sustainable biopolymer-based strategies for environmental applications, highlighting representative systems, their functional advantages, and the technical challenges that affect their performance and scalability. It should be noted that reported performance and functional advantages originate from studies conducted under heterogeneous experimental designs and validation conditions. Therefore, direct comparisons among systems are limited, and results should be interpreted with consideration of methodological variability, operational parameters, and technology readiness level.

The advances described in this section demonstrate that the development of sustainable biopolymers has moved beyond the simple substitution of conventional materials, evolving toward the design of highly functionalized, hybrid, and adaptive systems capable of addressing complex environmental challenges. However, despite progress in efficiency, selectivity, and the diversification of applications, critical limitations persist regarding operational stability, regenerability, scalability, reproducibility, and the economic viability of these emerging technologies.

These gaps highlight the need to analyze biopolymers not only from the perspective of technical performance, but also in terms of their integration within models of circularity, technological scaling, and real-world implementation. From this perspective, the following section examines the role of biopolymers within the circular economy, evaluating their end-of-life pathways, technology readiness levels (TRLs), and the challenges associated with transitioning from laboratory research to pilot-scale and industrial environmental applications.

## 7. Recent Case Studies with Outstanding Performance of Biopolymers in Environmental Applications

To support the trends discussed previously with recent experimental evidence, this section presents representative case studies that report outstanding quantitative performance of biopolymeric materials in relevant environmental applications, selected according to criteria including high adsorption or separation performance, structural stability, regenerability, advanced functional design, and demonstrated operational feasibility.

A recent experimental study reported three-dimensional microspheres based on hydrothermally modified chitosan with attapulgite (ATP), immobilized within a calcium chloride (CaCl_2_) crosslinked alginate matrix, referred to as hydrothermal chitosan–attapulgite composite embedded in alginate microspheres (CATP@SA3), for the removal of Pb(II) ions from water. The material exhibited a high density of active functional groups (–NH_2_, –OH, and –COOH), favoring chemisorption and ion-exchange mechanisms primarily. A maximum adsorption capacity of 1081.36 mg/g was obtained using the Langmuir model, a value markedly higher than those reported for conventional chitosan hydrogels. In addition, the adsorbent maintained more than 70% efficiency after five regeneration cycles using sodium ethylenediaminetetraacetate (Na_2_EDTA), demonstrating good structural stability and strong potential for reuse in aqueous systems contaminated with heavy metals [159].

Complementarily, a recent example of multifunctional biopolymeric cryogels with outstanding dye-removal performance reported the development of magnetic cryogenic beads based on chitosan, kaolinite, cellulose nanofibrils (CNF), and iron oxide nanoparticles (Fe_3_O_4_), synthesized through a green freeze-gelation process and genipin crosslinking. The resulting macroporous architecture, characterized by interconnected pores of 100–250 μm and a high specific surface area of 230 m^2^/g, promoted rapid methylene blue diffusion and highly efficient adsorption under neutral conditions. The material achieved a maximum adsorption capacity of 812 mg/g, as determined by the Langmuir model (R^2^ = 0.997). It exhibited extremely fast equilibrium (95% in 22 min) with pseudo-second-order kinetics (R^2^ > 0.99), confirming a chemisorption-dominated mechanism. In situ incorporation of Fe_3_O_4_ conferred excellent magnetic responsiveness, reaching a saturation magnetization (M_s_) of 20 emu/g, enabling recovery of more than 99% of the adsorbent in less than 35 s using an external magnet. Moreover, the beads retained over 90% of their efficiency after 6 adsorption–desorption cycles, demonstrating high structural stability, regenerability, and operational feasibility for sustainable wastewater treatment [160].

Continuing with highly porous materials, regenerable aerogels based on cellulose nanofibrils combined with sodium alginate and polyglutamic acid were developed via a green one-pot method and evaluated in both monolithic and bead forms for the removal of Pb(II), Zn(II), and Cu(II) from water. The bead configuration exhibited higher efficiency, achieving maximum adsorption capacities of 171.7 mg/g for Pb(II), 100.0 mg/g for Zn(II), and 142.0 mg/g for Cu(II). The material maintained structural stability after 20 regeneration cycles using citric acid, demonstrating high reusability and sustained effectiveness in heavy metal capture [161].

Expanding the approach toward hybrid configurations with embedded nanomaterials, macroscopic beads of crosslinked alginate were developed incorporating magnetic ferrite nanoparticles and two-dimensional graphene oxide (GO) composites decorated with Fe_3_O_4_ for the removal of Cr(III), Ni(II), and Cu(II) from aqueous solutions. The immobilization of these active phases within the biopolymeric matrix improved handling of the adsorbent. It promoted additional interactions with the carboxylate groups of alginate, leading to adsorption capacities superior to those of the free nanomaterials. Although the time required to reach equilibrium was longer due to internal diffusion limitations, the beads exhibited greater structural stability and strong potential for application in fixed-bed systems, making them an effective strategy to combine high removal efficiency with easy recovery and operational feasibility in the treatment of heavy-metal-contaminated waters [162].

A recent study developed a composite membrane based on chitosan (CS) and polyvinyl alcohol (PVA), incorporating Zeolitic Imidazolate Framework-8 (ZIF-8) nanoparticles as a selective phase on a porous polyvinylidene fluoride (PVDF) support. The support was fabricated via non-solvent-induced phase separation (NIPS) and optimized via a modified immersion precipitation process followed by spin coating (MIPS-SC), a strategy introduced by the authors to improve support structure and active-layer integration simultaneously. This architecture enabled homogeneous distribution of ZIF-8 within the CS/PVA layer, generating selective transport pathways that promoted molecular sieving and significantly enhanced the rejection of organic dyes and metal ions compared to membranes without metal–organic frameworks (MOFs). The CS/PVA/ZIF-8 membrane achieved a water permeate flux of 342 L/m^2^·h·bar, while maintaining high selectivity and structural stability, confirming the synergistic effect of the microstructured PVDF support and the porous ZIF-8 phase on the performance of sustainable membranes for water purification [163].

Another recent experimental study developed biodegradable nanocomposite films based on potato starch reinforced with two-dimensional titanium carbide nanoplatelets (MXene) of the Ti_3_C_2_T_x_ type, prepared by solution casting, achieving simultaneous improvements in mechanical and barrier properties. The incorporation of 10 wt% MXene markedly increased the Young’s modulus from 456.5 to 1923.6 MPa and the tensile strength from 9.9 to 19.1 MPa, attributable to strong interfacial interactions and the orientation of the nanoplatelets within the polymer matrix. More importantly, the films exhibited an extraordinary reduction in water vapor permeability (92.9%) and oxygen permeability (74.0%), indicating the formation of highly tortuous pathways that effectively limit molecular diffusion. Despite the inorganic reinforcement, the films retained their biodegradability, completely disintegrating in soil after 6 weeks, confirming their strong potential as sustainable advanced barrier materials [164].

Finally, a recent study developed a sustainable hybrid adsorbent, crystalline nanocellulose functionalized with N-[3-(trimethoxysilyl)propyl]ethylenediamine (SPEDA@nanocell), for the removal of the textile dye Reactive Yellow 2 from water. The chemical modification introduced protonatable amine groups that favored mechanisms dominated by electrostatic attraction under acidic conditions (optimal pH = 2). The material achieved a maximum adsorption capacity of 112.6 mg/g at 45 °C, was well fit by the Liu model (adjusted R^2^ ≈ 0.9999), and exhibited endothermic behavior (ΔH° = 23.44 kJ/mol), consistent with predominantly physical interactions. Despite its very low specific surface area (<1 m^2^/g), the adsorbent displayed kinetics well described by a fractal-PFO model and high structural stability, maintaining its adsorption capacity after five regeneration cycles with recoveries exceeding 96% using organic solvents. This work demonstrates that organosilane–amine functionalization of nanocellulose can compensate for textural limitations and generate highly efficient adsorbents for anionic dyes in complex aqueous systems [165].

Overall, the recent case studies presented demonstrate that the high performance of biopolymers in environmental applications is closely linked to advanced structural design strategies, targeted chemical functionalization, and integration with nanomaterials and metal–organic frameworks (MOFs). These investigations show that configurations such as composite hydrogels, magnetic cryogels, regenerable aerogels, hybrid beads, and sustainable mixed membranes can achieve efficiencies comparable to, or even higher than, those of conventional technologies, while simultaneously meeting sustainability and reusability criteria.

In addition, emerging evidence suggests that hybrid architectures combining starch, chitosan, and natural gums, particularly in formats such as microspheres and cryogels, represent a promising design direction for next-generation environmental biopolymers. These multicomponent systems can exploit synergistic interactions among polysaccharide matrices, improving mechanical stability, adsorption efficiency, and regeneration capacity while enabling tunable structural properties and process adaptability. Such hybrid configurations are increasingly recognized as scalable platforms with strong potential for advanced water treatment and pollutant removal applications.

Nevertheless, direct comparisons across studies should be interpreted with caution, as differences in experimental conditions, test matrices, and evaluation protocols can lead to significant variations in reported performance. Furthermore, the results highlight the importance of evaluating regenerability, stability in real matrices, and operational feasibility at larger scales, all of which are directly related to the principles of the circular economy. In addition, realistic circular implementation requires considering material end-of-life pathways and the potential recovery or valorization of captured contaminants as secondary resources. From this perspective, the following section further explores integrating technological performance, scalability, and circular strategies to support the sustainable implementation of these materials in real-world contexts.

## 8. Circular Economy, Scalability, and Future Perspectives

The transition of biopolymers toward a truly circular economy requires consideration of both end-of-life material pathways and a comprehensive assessment of their environmental impacts. In this context, biodegradation, mechanical recycling, and composting constitute complementary options that enable reducing waste accumulation and reintegrating materials into the productive cycle, provided that products are designed from the outset to facilitate recovery at the end of their service life. Recent life cycle assessment (LCA) studies have documented that biopolymers can have lower environmental footprints than conventional petroleum-derived plastics, particularly when scenarios that incorporate credits for material or energy recovery are considered. However, the heterogeneity of methodologies and available data continues to pose significant methodological challenges for consistent comparisons among material alternatives. On the other hand, the valorization of recovered contaminants, such as heavy metals or organic compounds, represents an opportunity to generate valuable secondary resources within circular systems, reducing the need for virgin inputs and strengthening the integration between environmental treatment and the circular economy [118,166].

The scalability and industrialization of technologies based on sustainable biopolymers involve not only scientific advances but also the evaluation of technology readiness levels (TRLs), production costs, and regulatory barriers. Currently, many developments in functionalized biopolymers and hybrid materials remain at intermediate stages of technological maturity, with pilot-scale implementations demonstrating their potential for integration into water and soil treatment processes. However, they still face limitations in recovery infrastructure and regulatory frameworks that do not always distinguish between bio-based materials and conventional polymers. Cost–benefit comparisons with conventional materials such as activated carbon, zeolites, and synthetic membranes highlight the importance of designing more efficient production strategies, less resource intensive synthesis processes, and regulatory frameworks that recognize comprehensive sustainability criteria, particularly when considering lifecycle performance, regeneration capacity, and operational durability, which often offset higher initial material costs and can significantly accelerate the industrial adoption of biopolymer based solutions [167].

Looking ahead, research on sustainable biopolymers must focus on overcoming critical gaps that currently limit their practical implementation. Among these, key priorities include the development of materials with advanced selectivity for emerging contaminants, standardized evaluation protocols for real environmental matrices, and effective regeneration strategies that maintain functional performance across multiple use cycles. Moreover, integrating these approaches with public policies on the bioeconomy and the circular economy is essential to align scientific knowledge generation with regulatory and market incentives, thereby fostering more resilient and sustainable productive systems. Strengthening cooperation among academia, industry, and regulatory bodies will enable the definition of clear pathways from laboratory research to real-scale applications, maximizing the potential of biopolymers as advanced, biodegradable, and socially responsible environmental solutions [118,166].

In summary, sustainable biopolymers represent a strategic technological platform with genuine potential to transform traditional environmental remediation schemes toward more efficient, regenerative, and circularity-aligned models. Nevertheless, their consolidation will depend on the convergence of material innovation, validation under real operational conditions, and the feasibility of industrial-scale implementation—dimensions that still require coordinated efforts.

The integration of these materials into circular value chains will not only help reduce the environmental impacts associated with persistent contaminants and waste but also support the circular economy. However, it will also open opportunities for the valorization of recovered resources and the development of environmentally responsible technologies with tangible social impact. This scenario positions biopolymers not as isolated substitutes but as key components of hybrid and sustainable solutions that will shape the future evolution of environmental engineering.

**Figure 3:**
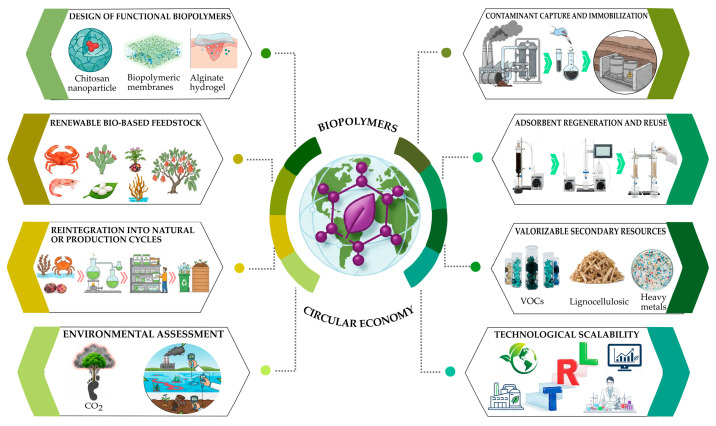
Circular economy conceptual framework applied to the functional life cycle of biopolymers in environmental remediation.

To schematically integrate the concepts addressed in this section, Figure 3 presents a conceptual framework describing the functional life cycle of biopolymers within a circular economy approach applied to environmental remediation. The scheme illustrates the sequence linking the renewable origin of materials, their transformation into functional biopolymers, their application in contaminant capture or immobilization across different environmental compartments, the recovery and regeneration of the adsorbent material, and the end-of-life pathways that enable reintegration into natural or productive cycles. This representation visually synthesizes the interaction between material sustainability, environmental performance, and circularity, reinforcing the role of biopolymers as integrated platforms within sustainable environmental engineering strategies.

## 9. Conclusions

Overall, the evidence synthesized in this review highlights key conceptual and practical insights into the role of sustainable biopolymers in environmental engineering.

Sustainable biopolymers constitute structurally tunable multifunctional platforms whose environmental performance depends on molecular composition, architecture, and degree of functionalization. When rationally engineered into optimized configurations such as hydrogel membranes, beads, aerogels, and hybrids, they can achieve efficiencies comparable to conventional materials while offering advantages in sustainability and regeneration potential. However, their main technological limitations relate to mechanical stability, selectivity in complex matrices, recovery efficiency, and scalability. In this context, hybridization strategies, targeted functionalization, and green synthesis routes represent the most promising directions for improving performance and applicability. Real-scale validation, methodological standardization, and techno-economic assessment are also decisive for accelerating industrial implementation.

Recent advances show a clear transition toward functionalized biopolymers, hybrid materials with inorganic phases, and green synthesis technologies that enhance environmental performance and broaden application potential. Nevertheless, the lack of methodological standardization, limited validation under real operating conditions, and economic and regulatory barriers remain major challenges for large-scale consolidation and objective comparison with established conventional technologies. It is therefore essential to distinguish between laboratory-scale demonstrations and systems that have reached pilot or industrial validation, since performance observed under controlled conditions does not necessarily translate directly to real-scale applications.

## Figures and Tables

**Table 1:** Comparison of the main forms of biopolymer application in environmental engineering (advantages, limitations, and performance examples).

Application Form	Typical Biopolymers	Main Environmental Applications	Operational Advantages	Technical Limitations	General Performance Examples	Reference
Hydrogels	Chitosan	Adsorption and removal of heavy metals and organic dyes from contaminated wastewater.	High density of –NH_2_ functional groups; strong electrostatic and coordination affinity for contaminants; high chemical reactivity; easy shaping and processing into diverse functional architectures.	pH sensitivity; low solubility requiring acidic media for dissolution; excessive swelling in aqueous media; low mechanical strength and structural stability without crosslinking or reinforcement.	High adsorption efficiency for multiple heavy metals, including Pb(II) (up to 1331.6 mg/g), Cd(II) (up to 656.0 mg/g), Cu(II) (up to 298.6 mg/g), Ni(II) (up to 243.1 mg/g), and Zn(II) (up to 236.0 mg/g); performance enhanced by hybridization, crosslinking strategies, and multifunctional composite network design, with demonstrated applicability in dye removal systems.	[40,78,79]
	Alginate	Removal of heavy metals and cationic dyes from contaminated wastewater; immobilization and stabilization of contaminants in hydrogel matrices.	Mild ionic crosslinking (Ca^2+^–alginate networks); strong electrostatic affinity for metal cations and cationic dyes; high porosity and water permeability; biocompatibility and low toxicity; easy functionalization and hybridization with carbon materials, clays, and nanofillers.	Low intrinsic mechanical strength; sensitivity to ionic strength and pH; structural instability under high salinity and long-term operation without reinforcement; swelling-induced loss of structural integrity in aqueous systems.	Moderate to high adsorption performance in composite alginate-based hydrogels, with Cu(II) up to 26.5 mg/g and Ni(II) up to 41.81 mg/g in sustainable sodium alginate (SA) systems; high-performance composite networks reaching Pb(II) up to 664.6 mg/g, Cu(II) up to 150.3 mg/g, and Cr(VI) up to 133.7 mg/g; and efficient dye removal in hybrid hydrogels, with methylene blue (MB) adsorption up to 136.98 mg/g and eriochrome black T (EBT) up to 66.66 mg/g, and removal efficiencies of up to 84.9%.	[80,81,82]
	Cellulose; cellulose derivatives; nanocellulose-based functional materials.	Removal of heavy metals and dyes; immobilization of organic contaminants in hydrogel matrices.	High adsorption efficiency after functionalization; large specific surface area (nanocellulose); mesoporous structure; abundant functional groups; strong chelation and ion-exchange capacity; chemical and structural stability; easy functionalization and composite engineering.	Low affinity without chemical modification; need for functionalization/composite engineering; synthesis complexity; higher cost of nanocellulose-based systems.	Significant performance improvement after functionalization, with adsorption capacities reaching Cu(II) 200.44 mg/g and Zn(II) 192.28 mg/g in functionalized CMC hydrogels; additionally, Cd(II) up to 147.7 mg/g, Hg(II) up to 88.62 mg/g, and Pb(II) up to 163.89 mg/g in cellulose-based composite hydrogels; furthermore, high adsorption capacity for cationic dyes (methylene blue) in nanocellulose-based hydrogels, with surface areas up to 697 m^2^/g and pseudo-second-order kinetics; high structural and regeneration stability over ≥5 adsorption–desorption cycles.	[83,84,85]
	Starch (native and modified).	Adsorption of heavy metals and emerging organic contaminants in water; hydrogel matrices and functional supports for composite and hybrid systems.	High availability and low cost; renewable and biodegradable matrix; high density of hydroxyl groups enabling chemical functionalization; tunable hydrophilicity; excellent swelling behavior; suitability for macromolecular engineering through crosslinking, grafting, blending, and composite formation.	Poor intrinsic mechanical stability; structural degradation in aqueous media; water solubility of native starch; limited long-term durability; low resistance to repeated operational cycles without crosslinking or composite reinforcement; scalability constraints for complex hydrogel architectures.	High adsorption capacities for Pb(II), Cd(II), and Cu(II) (≈150–500 mg/g in modified starch hydrogels and composites); organic contaminant removal efficiencies frequently exceeding 80–95% in crosslinked and hybrid networks; improved mechanical integrity and regeneration behavior, with stable adsorption–desorption performance over ≥4–6 cycles; adsorption efficiency and durability strongly controlled by macromolecular engineering, functional group density, crosslinking degree, and network architecture in composite and nanocomposite systems.	[33,36]
Cryogels	Chitosan; chitosan–alginate; chitosan–cellulose.	Adsorption of heavy metals and dyes under continuous-flow conditions; dynamic water treatment in fixed-bed and column systems.	Interconnected macroporous three-dimensional network; high permeability and low hydraulic resistance; efficient convective mass transfer; rapid diffusion kinetics; suitability for continuous-flow operation; reusability in dynamic treatment systems; structural continuity favoring low pressure drop.	Lower specific surface area compared to aerogels and nanostructured adsorbents; more complex cryostructuring synthesis routes; mechanical fragility without crosslinking or composite reinforcement; limited structural load-bearing capacity in large-scale column systems without mechanical support.	High dynamic removal efficiencies for Pb(II), Cd(II), and organic dyes under continuous-flow conditions; stable performance in column operation with maintained adsorption efficiency over multiple adsorption–desorption cycles; enhanced dynamic stability, permeability, and mass-transfer efficiency in reinforced chitosan–alginate and chitosan–cellulose cryogel composites; performance strongly governed by pore architecture, cryostructuring conditions, crosslinking degree, and composite reinforcement strategy.	[86,87,88,89]
	Cellulose; nanocellulose; composite cellulosic cryogels.	Adsorption of organic and inorganic contaminants; structural support matrices for hybrid systems and dynamic column-based water treatment processes.	High structural and mechanical stability; controlled macroporous architecture; excellent chemical robustness; good compatibility with surface functionalization and chemical grafting; tunable surface chemistry; suitability for continuous-flow operation; low deformation under hydraulic stress; effective mass transfer in dynamic systems.	Low intrinsic adsorption affinity without chemical modification or surface functionalization; limited selectivity in native form; costs and scalability constraints associated with nanocellulose production and processing; need for functionalization to achieve high-performance adsorption.	Significant enhancement in heavy metal and dye removal after chemical functionalization and surface modification; high operational and mechanical stability in dynamic systems; stable adsorption performance over multiple adsorption–desorption cycles; improved dynamic performance in hybrid and composite cellulosic cryogel networks; adsorption efficiency and durability strongly governed by surface chemistry, functional group density, macroporous architecture, and composite reinforcement strategy.	[90,91,92,93]
Aerogels	Cellulose; nanocellulose; chitosan; alginate; composite and hybrid biopolymeric aerogels.	Adsorption of heavy metals, dyes, pharmaceuticals, oils, and organic contaminants; water purification and remediation; filtration systems; environmental separation processes.	Very high porosity and low density; interconnected 3D porous networks; high adsorption capacity; tunable surface chemistry via functionalization; compatibility with hybrid and composite reinforcement strategies; suitability for multifunctional environmental remediation systems.	Mechanical fragility; high processing and drying costs (supercritical CO_2_ drying or freeze-drying); structural collapse without reinforcement; limited mechanical stability under continuous-flow and hydraulic stress conditions; scalability constraints; need for chemical crosslinking and composite reinforcement for real-system applications.	High adsorption performance in functionalized and composite aerogels, with reported capacities for Pb(II) up to 643.62 mg/g and Cd(II) up to 250.31 mg/g in advanced hybrid systems; high removal efficiencies for dyes and pharmaceuticals in biopolymeric and hybrid aerogels, including diclofenac sodium up to 605.87 mg/g, methylene blue up to 289.6 mg/g, and methyl blue up to 1369.1 mg/g; excellent performance in oil/water separation and contaminant removal (e.g., toluene 170 mg/g, xylene 200 mg/g); improved regeneration and reuse, with stability maintained over multiple adsorption–desorption cycles in reinforced and composite systems; adsorption efficiency and durability strongly governed by pore architecture, surface functionalization, and hybridization strategy.	[69,94,95,96]
Functionalized beads and particles	Alginate; chitosan; alginate–chitosan; crosslinked polysaccharide-based composites.	Adsorption of heavy metals and dyes; immobilization of active phases (metal oxides, catalysts, enzymes, functional fillers); wastewater treatment in batch and continuous-flow systems; fixed-bed, column, and packed-bed filtration processes.	Low-cost, sustainable materials; simple, scalable fabrication; chemical and ionic crosslinking versatility; good mechanical stability after crosslinking; easy separation and recovery from aqueous media; compatibility with packed-bed and column reactors; tunable surface functionality through chemical modification and composite reinforcement.	Limited internal diffusion and mass-transfer resistance; lower specific surface area than aerogels and cryogels; pore blockage and diffusion-controlled transport; reduced adsorption kinetics in dense beads; gradual efficiency loss after multiple adsorption–desorption cycles without appropriate regeneration strategies.	High removal efficiencies for Pb(II), Cu(II), Cd(II), and diverse dyes in alginate-, chitosan-, and alginate–chitosan-based crosslinked beads; stable adsorption performance in chemically and ionically crosslinked systems; effective immobilization of active phases enabling multifunctional adsorption and catalytic behavior; suitability for long-term operation in batch and continuous systems, with adsorption efficiency and durability strongly governed by crosslinking density, bead porosity, surface functional group availability, and diffusion-controlled transport mechanisms.	[97,98,99,100]
	Chitosan; alginate; cellulose derivatives; polysaccharide-based composite beads.	Adsorption of heavy metals and dyes; removal of organic contaminants (oils, antibiotics); immobilization of active phases (metal oxides, catalysts, enzymes, functional fillers); wastewater treatment in batch and continuous-flow systems; fixed-bed, column, and packed-bed filtration processes.	High selectivity toward target contaminants; fast adsorption kinetics; easy separation and recovery (including magnetic separation); high removal efficiency; good regenerability and reusability; multifunctionality enabled by composite and hybrid bead architectures.	Higher synthesis complexity; increased production costs; potential leaching of active phases; structural heterogeneity; long-term stability strongly dependent on composite design, crosslinking strategy, and immobilization efficiency.	Enhanced adsorption performance and selectivity in composite and magnetic biopolymeric beads, with reported adsorption capacities of 129.6 mg/g for Cu(II) in Fe_3_O_4_–chitosan beads (corresponding to 617.1 mg/g based on chitosan content), rapid adsorption kinetics with equilibrium achieved within 10 min, and efficient magnetic separation within 30 s under low magnetic fields; strong reusability enabled by effective regeneration using chelating agents (e.g., EDTA), with stable performance over multiple adsorption–desorption cycles; overall efficiency and durability governed by bead architecture, surface functionalization, composite structure, and immobilization strategy.	[70,101]
Sustainable membranes	Chitosan; cellulose; nanocellulose; PLA; PHA/PHB; composite and hybrid biopolymeric membranes.	Ultrafiltration and nanofiltration of water; removal of heavy metals, dyes, organic matter, and emerging contaminants; desalination and reverse osmosis (RO) support layers; air purification (VOCs and particulate matter [PM]); ambient air filtration systems.	Renewable and bio-sourced origin; tunable surface chemistry through functionalization; good contaminant affinity and selectivity; lower environmental footprint; compatibility with electrospinning, phase inversion, and thin-film composite fabrication; integration into continuous and modular separation processes; suitability for hybrid and multilayer membrane architectures.	Lower chemical, thermal, and mechanical stability compared to synthetic membranes; fouling susceptibility; swelling in aqueous environments; need for reinforcement, crosslinking, or hybridization; durability limitations under high pressure and harsh operating conditions; scalability and long-term operational stability constraints.	Effective rejection of dyes, heavy metals, organic matter, and airborne particulates in sustainable biopolymeric membrane systems; improved selectivity, permeability, and antifouling behavior through nanoreinforcement (nanocellulose, inorganic fillers) and surface functionalization; enhanced separation efficiency and durability in composite and thin-film biopolymer-supported membranes; multifunctional performance in water purification, reverse osmosis support layers, and air filtration applications, with membrane efficiency and stability strongly governed by polymer chemistry, membrane architecture, and hybridization strategy.	[29,102,103,104]
Biodegradable films and coatings	Chitosan; starch; cellulose and nanocellulose; proteins (gelatin, whey, soy protein); PLA; PHA/PHB; biocomposites and hybrid biopolymeric systems.	Sustainable and active packaging (especially food and pharmaceutical packaging); protective coatings for perishable and sensitive materials; plastic waste reduction and substitution of fossil-based plastics; functional barrier systems against oxygen, moisture, light, and microbial contamination; smart packaging and responsive coating technologies.	Renewable and bio-sourced origin; intrinsic biodegradability and compostability; tunable barrier properties through formulation, blending, and functionalization; incorporation of bioactive agents (antimicrobials, antioxidants, antifungals); compatibility with circular-economy models; valorization of agro-industrial by-products and residues; adaptability to industrial processing techniques (casting, extrusion, coating, multilayer lamination, electrospinning).	High moisture sensitivity due to hydrophilic polymer backbones; lower mechanical strength and thermal resistance compared to fossil-based polymers; aging, brittleness, and plasticizer migration; limited resistance to harsh processing and storage conditions; requirement for reinforcement strategies (nanocomposites, multilayer architectures, hybrid coatings, crosslinking) to achieve industrial performance standards and long-term stability.	Significant improvement of oxygen and water-vapor barrier properties through nanoreinforcement (nanocellulose, layered silicates, inorganic fillers) and multilayer architectures; enhanced antimicrobial and antioxidant functionality via incorporation of natural extracts, essential oils, and bioactive compounds; effective shelf-life extension of packaged products; optimized mechanical, thermal, and barrier performance through hybrid film systems, nanocomposites, and multifunctional coating designs, enabling high-performance biodegradable alternatives to conventional plastic packaging.	[75,76,77,105,106]

**Table 2:** Emerging technologies based on sustainable biopolymers for environmental applications: approaches, advantages, and current challenges.

Emerging Technological Approach	Representative Biopolymeric Systems	Main Environmental Applications	Key Functional Advantages	Main Current Challenges	Reference
Nanocellulose functionalization—MOFs	Cellulose nanofibril (CNF) aerogel with Co–MIL-53(Fe) MOF grown in situ and structured by directional freezing; cellulose aerogel modified with Zeolitic Imidazolate Framework-8 (ZIF-8) and polyethyleneimine (PEI).	Catalytic degradation of antibiotics (tetracycline) via peroxymonosulfate (PMS) activation; selective CO_2_ capture.	Stable immobilization of MOF in a 3D network avoiding powder use; ordered porous structure improving mass transfer; degradation of tetracycline hydrochloride (TC) up to ~95% in 2 h; increase in surface area (153.1 m^2^/g) and CO_2_ capacity (9.26 mmol/g); evident mechanical improvement after modification.	Multistep synthesis (MOF growth + aerogel assembly); dependence on specialized drying processes; need for uniform structural control of MOF within the matrix.	[120,132,133]
Nanocellulose functionalization—metallic nanoparticles/oxides	CNF nanocellulose or cellulose nanocrystals (CNC) functionalized with metal oxide nanoparticles such as zinc oxide (ZnO), titanium dioxide (TiO_2_), copper oxide (CuO), and iron oxide (Fe_3_O_4_), forming nanocomposites via ex situ or in situ synthesis.	Removal of aquatic contaminants (heavy metals, dyes, organic compounds) via adsorption/catalysis and photocatalysis under irradiation.	Nanocellulose provides a high surface area and functional groups that improve nanoparticle dispersion and anchoring; metal oxides provide photocatalytic activity and enhanced adsorption capacity; the combination favors capture and degradation of contaminants.	Uniform control of nanoparticle distribution on nanocellulose; stability and recovery of the nanocomposite in real processes; possible nanoparticle aggregation and synthesis costs.	[134,135,136]
Nanocellulose functionalization—clays and nanoreinforcements	Nanocellulose (CNF/CNC) reinforced with montmorillonite (MMT) in hybrid matrices; nanocrystalline cellulose systems combined with bentonite reported as adsorbents.	Heavy-metal adsorption demonstrated in CNC–bentonite matrices; interest in sustainable hybrid materials for water treatment.	Incorporation of clays as functional inorganic phases within the nanocellulose network; structural improvement and interfacial compatibility in CNF–MMT systems; bentonite provides adsorptive capacity in CNC–bentonite systems.	Limited experimental evidence for CNF–clay systems in direct remediation; predominance of structural studies over environmental applications.	[123,134,137,138]
Energy-assisted green processes (ultrasound, microwaves)	Films and matrices based on biopolymers (modified starch, plant cellulose, polysaccharides) treated by ultrasound, microwaves, or combined ultrasound (US) and microwave (MW) processes.	Improvement of biodegradable materials with indirect potential in sustainable applications.	Ultrasound and microwaves induce morphological and structural changes that increase mechanical strength, homogeneity, and barrier properties; physical modification without chemical reagents; ultrasonic cavitation alters structure and macromolecular properties.	Evidence mainly focused on materials/films; there is a lack of direct validation in real environmental remediation systems.	[139,140,141]
Ionic liquids (ILs) for biopolymer synthesis and modification	Cellulose, chitosan, starch, and other biopolymers processed/dissolved or functionalized in ionic liquid media (imidazolium, cholinium, phosphonium).	Preparation of modified biopolymeric materials with potential for adsorption, membranes, and sustainable catalytic supports.	High capacity to dissolve biopolymers without prior derivatization; more efficient and homogeneous chemical modification; green alternative to volatile organic solvents.	High cost and complex recovery of ILs; potential toxicity depending on IL type; limited industrial scalability; biopolymer stability after regeneration.	[142,143,144,145]
Natural deep eutectic solvents (NADES)	NADES systems based on choline, organic acids, and natural compounds are applied to lignocellulosic biomass fractionation and lignin extraction.	Sustainable valorization of residual biomass; production of functional lignocellulosic fractions with potential for adsorbent materials and environmental processes.	High selective solubilization capacity for lignin; biodegradable and renewable-origin solvents; more sustainable processes than conventional solvents.	High viscosity limiting mass transfer; complex solvent recovery; stability and scalability are still under development.	[146,147,148,149]
Smart biopolymer-based materials (responsive hydrogels)	Chitosan and natural polysaccharide hydrogels designed as pH- and chemically responsive materials.	Removal of aquatic contaminants (heavy metals, dyes) through stimulus-dependent selective adsorption.	Reversible response to pH changes; swelling control; high affinity for contaminants; biodegradable and sustainable matrices.	Limited mechanical stability; partial regeneration; variable performance in real matrices.	[13,150,151,152]
Environmental biosensors based on biopolymers and composites	Films and matrices of chitosan, cellulose, alginate, and biocomposites with conductive nanoparticles or immobilized enzymes.	Detection of environmental contaminants (heavy metals, pesticides, organic compounds) in water and soils.	Biocompatibility and biodegradability; high chemical affinity for biomolecule immobilization; enhanced sensitivity in hybrid composites.	Limited biomolecule stability; interference in real matrices; reduced lifetime; need for frequent calibration.	[130,153,154]
Hybrid and complementary approaches versus conventional materials	Biopolymers combined with inorganic phases such as biochar, metal oxides, and nanomaterials form hybrid bio–inorganic composites.	Removal of contaminants in water (metals, organics, emerging compounds) using sustainable hybrid materials.	Synergy between the biopolymeric matrix and the inorganic phase improves structural stability and adsorptive functionality compared to individual materials.	Synthesis complexity, structural heterogeneity, long-term stability, and limited validation under real conditions.	[155,156,157,158]

## Data Availability

No new data were created or analyzed in this study. Data sharing is not applicable to this article.

## Abbreviations

The following abbreviations are used in this manuscript:

–COOH	Carboxyl group
–NH_2_	Amino group
–OH	Hydroxyl group
As	Arsenic
ATP	Attapulgite
Ca	Calcium ion
CaCl_2_	Calcium chloride
CATP@SA3	Hydrothermal chitosan–attapulgite composite embedded in alginate microspheres
Cd(II)	Cadmium ion
CMC	Carboxymethyl cellulose
CNC	Cellulose nanocrystals
CNF	Cellulose nanofibrils
Co–MIL-53(Fe)@CNF	Iron-based MIL-53 MOF, cobalt-doped and supported on cellulose nanofibrils
CO_2_	Carbon dioxide
Cr(III)	Chromium ion
Cr(VI)	Hexavalent chromium ion
CS	Chitosan
Cu(II)	Copper ion
CuO	Copper oxide
EBT	Eriochrome Black T
EDTA	Ethylenediaminetetraacetic acid
Fe_3_O_4_	Iron oxide (magnetite)
GO	Graphene oxide
Hg(II)	Mercury ion
ILs	Ionic liquids
LCA	Life Cycle Assessment
MB	Methylene blue
MIPS-SC	Modified Immersion Precipitation—Spin Coating
MMT	Montmorillonite
MOFs	Metal–organic frameworks
MW	Microwave
MXene	Two-dimensional titanium carbide (Ti_3_C_2_T_x_)
NADES	Natural deep eutectic solvents
Na_2_EDTA	Sodium ethylenediaminetetraacetate
NF	Nanofiltration
Ni(II)	Nickel ion
NIPS	Non-solvent induced phase separation
PEI	Polyethylenimine
PFAS	perfluoroalkyl and polyfluoroalkyl substances
PFO	Pseudo-first-order kinetic model
PHA	Polyhydroxyalkanoates
PLA	Polylactic acid
PM	Particulate matter
PMS	Peroxymonosulfate
PRISMA	Preferred Reporting Items for Systematic Reviews and Meta-Analyses
PRISMA–ScR	Preferred Reporting Items for Systematic Reviews and Meta-Analyses Extension for Scoping Reviews
PVA	Polyvinyl alcohol
PVDF	Polyvinylidene fluoride
pH	Potential of hydrogen
Pb(II)	Lead ion
RO	Reverse osmosis
SA	Sodium alginate
SC	Spin coating
SPEDA	N-[3-(trimethoxysilyl)propyl]ethylenediamine
TC	Tetracycline hydrochloride
TiO_2_	Titanium dioxide
TRL	Technology Readiness Levels
UF	Ultrafiltration
US	Ultrasound
UV	Ultraviolet radiation
VOCs	Volatile organic compounds
Zn(II)	Zinc ion
ZnO	Zinc oxide
ZIF-8	Zeolitic Imidazolate Framework-8
